# Elevated origin recognition complex subunit 6 expression promotes non-small cell lung cancer cell growth

**DOI:** 10.1038/s41419-024-07081-y

**Published:** 2024-09-30

**Authors:** Yong-hua Sang, Chun-ying Luo, Bing-tao Huang, Siyang Wu, Jian Shu, Chang-gong Lan, Fuquan Zhang

**Affiliations:** 1 Department of Cardiothoracic Surgery, The Second Affiliated Hospital of Soochow University, Suzhou, China; 2 Department of Pathology, Affiliated Hospital of Youjiang Medical University for Nationalities and Key Laboratory of Molecular Pathology in Tumors of Guangxi Higher Education Institutions, Baise, China; 3 Department of Thoracic Surgery, Binzhou Medical University Hospital, Binzhou, China; 4 Respiratory Intensive Care Unit, Affiliated Hospital of YouJiang Medical University for Nationalities, Baise, China; 5 Department of Thoracic and Cardiovascular Surgery, Taicang Affiliated Hospital of Soochow University The First People’s Hospital of Taicang, Taicang, China; 6 Department of Thoracic and Cardiovascular Surgery, The Second Affiliated Hospital of Nantong University, The First People’s Hospital of Nantong, Nantong, China

**Keywords:** Non-small-cell lung cancer, Targeted therapies

## Abstract

Exploring novel targets for non-small cell lung cancer (NSCLC) remains of utmost importance. This study focused on ORC6 (origin recognition complex subunit 6), investigating its expression and functional significance within NSCLC. Analysis of the TCGA-lung adenocarcinoma database revealed a notable increase in *ORC6* expression in lung adenocarcinoma tissues, correlating with reduced overall survival, advanced disease stages, and other key clinical parameters. Additionally, in patients undergoing surgical resection of NSCLC at a local hospital, *ORC6* mRNA and protein levels were elevated in NSCLC tissues while remaining low in adjacent normal tissues. Comprehensive bioinformatics analyses across various cancers suggested that ORC6 might play a significant role in crucial cellular processes, such as mitosis, DNA synthesis and repair, and cell cycle progression. Knocking down ORC6 using virus-delivered shRNA in different NSCLC cells, both primary and immortalized, resulted in a significant hindrance to cell proliferation, cell cycle progression, migration and invasion, accompanied by caspase-apoptosis activation. Similarly, employing CRISPR-sgRNA for ORC6 knockout (KO) exhibited significant anti-NSCLC cell activity. Conversely, increasing ORC6 levels using a viral construct augmented cell proliferation and migration. Silencing or knockout of ORC6 in primary NSCLC cells led to reduced expression of several key cyclins, including Cyclin A2, Cyclin B1, and Cyclin D1, whereas their levels increased in NSCLC cells overexpressing ORC6. In vivo experiments demonstrated that intratumoral injection of ORC6 shRNA adeno-associated virus markedly suppressed the growth of primary NSCLC cell xenografts. Reduced ORC6 levels, downregulated cyclins, and increased apoptosis were evident in ORC6-silenced NSCLC xenograft tissues. In summary, elevated ORC6 expression promotes NSCLC cell growth.

## Introduction

Lung cancer represents a substantial socioeconomic burden globally, causing a significant number of annual mortalities [1–3]. In the United States alone, there were over 220,000 new cases of lung cancer and 140,000 associated deaths in 2019 [1, 2]. It comprises two primary histological types: small cell lung cancer (SCLC) and non-small cell lung cancer (NSCLC) [1, 2]. NSCLC, constituting 85% of all lung cancer cases, is particularly prevalent, and lung adenocarcinoma (LUAD) is one of the most common NSCLC [1–4]. NSCLC treatment involves a multidisciplinary approach including surgery, radiation therapy, chemotherapy, targeted therapy, immunotherapy, and precision medicine [5, 6]. Patients diagnosed with NSCLC often present at advanced stages or experience relapse following curative-intent surgery, resulting in a challenging prognosis [7, 8].

Genes with abnormal expression/mutation and dysregulated signaling pathways play a critical role in the development, progression, and treatment resistance in NSCLC [9–13]. Current targeted therapies include agents targeting EGFR mutations (including gefitinib, erlotinib, and osimertinib), ALK rearrangements (including crizotinib, alectinib, and ceritinib), BRAF mutations (including dabrafenib, and trametinib), and MET alterations (including crizotinib and capmatinib) [9, 14–16]. However, for the majority of patients in advanced stages, the prognosis remains unsatisfactory [9, 14, 17, 18]. Exploring new molecular targets crucial for uncontrolled cell growth in NSCLC becomes imperative for improved therapeutic strategies to combat this disease [9–13].

DNA synthesis is a fundamental process necessary for the proliferation of NSCLC and other cancer cells [19–22]. NSCLC growth involves uncontrolled cell division and augmented cell cycle progression, and DNA synthesis allows cancer cells to replicate their DNA, enabling them to proliferate rapidly [19–22]. The origin recognition complex (ORC) is a highly conserved protein complex found in eukaryotic cells, essential for initiating DNA replication [23–25]. Comprising six distinct subunits (ORC1-ORC6), ORC binds to the origins of replication during the G1 phase of the cell cycle and recruits additional proteins to form pre-replication complex, which is pivotal for maintaining genome stability and ensuring accurate DNA replication [25–28]. ORC6, as part of ORC, is vital for DNA replication initiation [25–28]. Studies indicate that alterations in ORC6 expression or function may contribute to tumorigenesis and cancer progression in some certain cancers [29–33]. Its expression and functional significance within NSCLC have not been extensively studied thus far. In this study, we demonstrate that increased ORC6 expression promotes NSCLC cell growth in vitro and in vivo, establishing it as a promising and innovative therapeutic target.

## Materials and methods

### Reagents and antibodies

Puromycin, medium, serum, polybrene, and the RNA assay reagents as well as cell counting kit-8 (CCK-8) reagent and Trypan blue dye were purchased from Sigma-Aldrich (St. Louis, Mo). Fluorescence dyes, such as EdU (5-ethynyl-2′-deoxyuridine), TUNEL (terminal deoxynucleotidyl transferase dUTP nick end labeling), and JC-1, were acquired from Invitrogen Thermo-Fisher (Shanghai, China). All antibodies utilized in the research were obtained from Cell Signaling Tech (Shanghai, China).

### Cells

NSCLC cell line, A549, was procured from the American Type Culture Collection (ATCC, Manassas, VA). These cells were cultured in DMEM high-glucose medium supplemented with 10% FBS (fetal bovine serum). Additionally, primary human NSCLC cells (pNSCLC-1, pNSCLC-2, and pNSCLC-3), obtained from three different patients with written informed consent, as well as the human primary lung epithelial cells, were reported in our previous studies [34–36]. These cells were maintained in the specified medium conditions as described in the previous publications [35]. Routine checks were performed for mycoplasma and microbial contamination, STR (short tandem repeat) profiling, population doubling time, and cell morphology. Protocols of the use of primary human cells were reviewed and approved by the Ethics Committee of Soochow University (BR-2021-081), aligning with the principles outlined in the Declaration of Helsinki.

### Human tissues

A cohort comprising twenty (20) patients diagnosed with stage III-IV LUAD, aged between 44 to 81 years, and treated at the authors’ institutions, were included in this study. Each patient provided written informed consent before participation. Fresh tumor tissues and corresponding adjacent normal lung tissues were collected during surgery, and immediately preserved in liquid nitrogen for subsequent analysis. The handling of human tissues adhered to approved protocols reviewed by the Ethics Committee of Soochow University (BR-JY-2021-043), in compliance with the principles outlined in the Declaration of Helsinki.

### Immunohistochemistry

The paraffin-embedded human tissues or xenograft sections underwent a sequential series of baking, dewaxing, and hydration. Subsequently, they were washed using a 0.4% Triton X-100 in PBS (PBST) solution and then immersed in a 10% serum in PBST solution for 20 min to minimize any unspecific binding. Following this, the endogenous peroxidase activity was obstructed using hydrogen peroxide, and the primary antibody was applied, allowing an incubation period of 8 h to ensure proper binding to the target protein. Following the antibody application, a biotin-labeled IgG antibody was administered for 2 h, followed by an incubation period with streptavidin-horseradish peroxidase (HRP). Ultimately, the visualization of the targeted protein was accomplished using DAB staining.

### ORC6 silencing

shRNA experiments were conducted on NSCLC cells grown in complete medium with polybrene when reaching 60–65% confluence. Lentiviral particles expressing ORC6 shRNA, provided by Genechem (Shanghai, China), were introduced to the cultured cells at MOI = 15. The viral infection persisted for 48 h. Subsequently, puromycin-containing complete medium was added to the infected cells, and stable cells emerged following selection through 4–5 passages. Two distinct and verified shRNAs targeting non-overlapping sequences were employed: shORC6-1# and shORC6-2#. Control cells underwent infection with lentiviral particles containing a scramble control shRNA (“shC” [34–36]). Constant monitoring of *ORC6* mRNA and protein expression in the stable cells was performed. For in vivo studies, the shORC6-2# sequence or the shC sequence were inserted into a previously-described adeno-associated virus (aav) construct [34, 35] and virus was thereafter generated.

### CRISPR/Cas9-KO of ORC6

Gene knockout (KO) experiments were conducted on NSCLC cells grown in complete medium with polybrene at 50-60% confluence. These cells were infected with Cas9-expressing lentivirus [34–36], leading to the formation of stable cells following selection. The Cas9-expressing pNSCLC-1 cells were transduced with the lentivirus containing the CRISPR/Cas9-ORC6-KO puro-construct with verified sgRNA specific for ORC6, supplied by Genechem (Shanghai, China). Stable cells were established through puromycin treatment and were distributed into 96-well plate at single cell level. Sequencing of the *ORC6* gene around the targeted site confirmed *ORC6* gene abnormality, and its KO was further verified by Western blotting. Three stable single-cell-derived NSCLC-1 ORC6 KO colonies—koORC6-Slc1, koORC6-Slc2, and koORC6-Slc3—were successfully established, each exhibiting disrupted *ORC6* sequence and completely depleted ORC6 protein expression. Control cells underwent infection with lentivirus encoding the CRISPR/Cas9-empty control vector (“Ctrl”), as reported previously [34, 35].

### ORC6 overexpression

Gene overexpression experiments were conducted in NSCLC cells by culturing them in complete medium supplemented with polybrene and at 60% confluence. Lentivirus carrying the ORC6-expressing construct (GV369, Genechem [34, 35]) was used to infect the cells at MOI of 15. This construct encompassed the ORC6 cDNA sequence but has no tag, and the viral infection duration was 48 h. Subsequently, puromycin-containing complete medium was introduced to the infected cells, leading to the establishment of stable cell selections after five additional passages. The expression of ORC6 in these stable cells was consistently tested.

### Western blotting

Proteins were extracted from tissues and cells using the RIPA lysis buffer supplemented with protease inhibitors (Beyotime, Wuxi, China). The concentration of proteins was determined using a BCA Kit (Invitrogen, Shanghai, China). Subsequently, proteins were separated on 10–12.5% SDS-PAGE gels and transferred onto PVDF membranes (Millipore). After the blocking step, the membranes underwent overnight incubation with designated primary antibodies, succeeded by a 1 h exposure to secondary antibodies. The identification of target protein bands was accomplished through the utilization of the ECL system.

### Quantitative real-time polymerase chain reaction (qRT-PCR)

Cells and tissues were treated with TRIzol reagents (Takara, Otsu, Japan) for the extraction of total RNA. Following the determination of RNA concentration, reverse transcription into cDNA was performed using a Reverse Transcription Kit (Applied Biosystems, Foster City, CA). The qRT-PCR assays utilized the SYBR Green real-time PCR Kit (Takara, Tokyo, Japan) on the Bio-Rad CFX96 system (Bio-Rad, Hercules, CA). mRNA quantification followed the 2^−ΔΔCt^ method, with glyceraldehyde-3-phosphate dehydrogenase (GAPDH) examined as the endogenous control.

### EdU staining

NSCLC cells were seeded into 12-well plates, fixed, and permeabilized after 96 h. EdU and DAPI dyes were added, and fluorescence microscopy (Nikon, Shanghai, China) was utilized to visualize and calculate the percentage of EdU-positive cells in five random views. The average EdU fluorescence intensity for each individual nucleus in every microscopic field of view was calculated and its value was normalized to that in the control cells.

### p53BP1 staining

Cells cultured on glass coverslips were fixed in 4% paraformaldehyde and permeabilized with 0.5% Triton X-100. After blocking non-specific binding, cells were incubated with a primary p53BP1-specific antibody (Cell Signaling Tech) overnight at 4 °C and subsequently with a fluorescent secondary antibody. Nuclei were stained with DAPI. Following mounting, samples were analyzed using fluorescence microscopy.

### Comet assay

The Comet assay was carried out following the instructions of a commercialized Comet assay kit (Trevigen, Gaithersburg, MD).Cells were mixed with low melting point agarose and layered onto pre-coated slides, then lysed to expose nuclear DNA for 1 h at 4 °C. The slides underwent alkaline unwinding before electrophoresis at 21 V for 30 min, facilitating DNA migration. Post-electrophoresis, slides were neutralized, stained with ethidium bromide (EB), and analyzed under fluorescence microscopy.

### Transwell assays

NSCLC cells were added to the upper surfaces of “Transwell” chambers and allowed to migrate for 24 h. The cells migrated to the lower surface were fixed and stained with crystal violet. In cell invasion assays, Matrigel-coated “Transwell” chambers were utilized.

### TUNEL staining

NSCLC cells with genetic modifications or applied treatments were inoculated into 96-well plates and cultured for designated durations. Co-staining with DAPI and TUNEL was performed, and TUNEL-positive nuclei were captured under a fluorescence microscope (Nikon). The TUNEL ratio (% versus DAPI) was calculated in five random views, each containing total 500 nuclei.

### Annexin V-propidium iodide (PI) FACS

As described previously [34–36], NSCLC cells with the applied genetic modifications or treatments were inoculated into six-well plates. After culture for designated durations, cells were washed and incubated in 1× binding buffer supplemented with Annexin V-FITC and PI. Flow cytometry using a Beckman Coulter flow cytometer was employed for sorting. For sorting S-phase cells, cells were co-stained with EdU and PI.

### Other assays

including Cell Counting Kit-8 assaying of cell viability, PI-FACS (fluorescence-activated cell sorting) assaying of cell cycle progression, [^3^H] DNA incorporation assay, Caspase-3 activity assays, cytosol cytochrome-C ELISA, JC-1 (5,5′,6,6′-tetrachloro-1,1′,3,3′-tetraethylbenzimidazolylcarbocyanine iodide) measuring mitochondrial membrane potential were described in our previous studies [34–36]. Figure S1 listed un-cropped blotting images.

### Xenograft studies

The nude mice, comprising an equal number of male and female animals at 7–8 weeks of age and weighing between 18.1 and 18.6 g, were procured from the animal center of Soochow University. These mice were housed under standard conditions. To establish pNSCLC-1 xenografts, six million cells per mouse were subcutaneously inoculated into the flanks of the nude mice. Tumors formed within 3 weeks post inoculation, reaching volumes close to 100 mm^3^. Subsequently, the mice were randomly divided into two groups: ten mice received intratumoral injections of adeno-associated virus-packed ORC6 shRNA (“aav-shORC6-1#”), while the other ten received aav-packed scramble control shRNA (“aav-sh-scr”). The virus injections were administered twice, 72 h apart. Tumor volume was measured as previously described [34, 35]. The detailed procedures for tumor tissue TUNEL immunofluorescence assay were described elsewhere. All procedures involving animals were approved by the Institutional Animal Care and Use Committee (IACUC) and the Institute Animal Ethics Review Board of Soochow University.

### Statistical analyses

Statistical analyses were conducted with blinding of investigators to group allocation throughout all in vitro experiments. For normally distributed numeric data, values were presented as mean ± SD (standard deviation). To analyze differences among multiple groups, one-way analysis of variance (ANOVA) was employed, followed by Dunnett’s test using SPSS 23.0 (Chicago, CA). For comparisons between two specific groups, Student’s *t* test was utilized with EXCEL 2007. A significance level of *P* < 0.05 was considered statistically significant. All in vitro experiments were performed five times, yielding consistent results.

## Results

### Bioinformatics analysis reveals *ORC6* overexpression in NSCLC

**Fig. 1:**
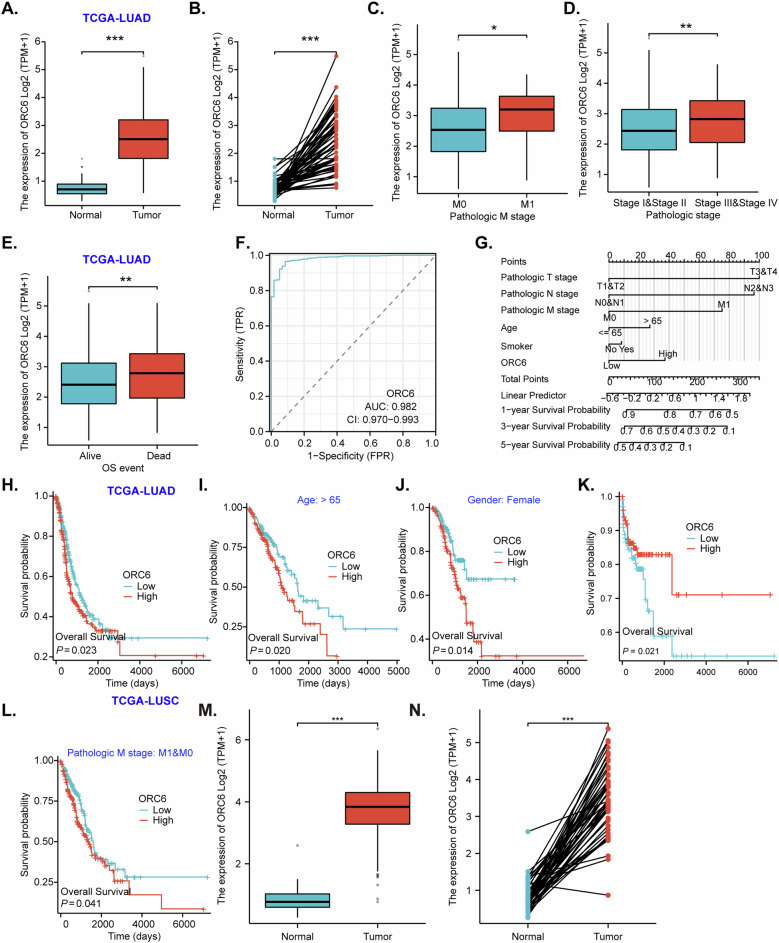
Bioinformatics analysis reveals *ORC6* overexpression in NSCLC. TCGA-LUAD cohort shows *ORC6* mRNA transcripts in the LUAD tissues (“Tumor”) and normal lung tissues (“Normal”) in specific patients (**A**–**E**); The receiver operating characteristic (ROC) curve analyzing *ORC6* expression and the potential predictive value of LUAD patients was shown (**F**). Clinical prediction model line chart shows the possible value of ORC6 overexpression in predicting prognosis of LUAD patients (**G**). The Kaplan Meier Survival curve of *ORC6*-low (blue) and *ORC6*-high (red) LUAD patients was presented (**H**). Subgroup Kaplan Meier Survival analyses, based on the different clinical features of the LUAD patients, were performed as well (**I**–**L**). TCGA-LUSC cohort shows *ORC6* mRNA transcripts in the LUSC tissues (“Tumor”) and normal lung tissues (“Normal”) (**M**, **N**); “TPM” stands for transcripts per million. “TRP” stands for “true positive rate”. “FRP” stands for “false positive rate” *P < 0.05, **P < 0.01, ***P < 0.001.

NSCLC is broadly categorized into three main subtypes, including adenocarcinoma (LUAD), squamous cell carcinoma (LUSC), and large cell carcinoma, among which LUAD is the most prevalent [3]. In The Cancer Genome Atlas (TCGA) database, the evaluation of *ORC6* expression in LUAD patients compared to normal lung tissue revealed significant differences. *ORC6* expression levels were significantly elevated in LUAD tissues (“Tumor”) compared to normal lung tissues (“Normal”) (Fig. 1A). *ORC6* overexpression in LUAD was further validated by conducting comparisons specifically within LUAD tissues and matched adjacent normal lung tissue tissues (Fig. 1B). The subgroup analyses further demonstrated that *ORC6* overexpression in human LUAD tissues correlated with higher M-stages (Fig. 1C) and higher pathological stages (Fig. 1D). Moreover, *ORC6* expression in LUAD tissues of deceased patients was significantly higher compared to that in alive LUAD patients (Fig. 1E).

ROC (Receiver Operating Characteristic) curve analysis was performed using TCGA-LUAD *ORC6* expression data, yielding an area under the curve (AUC) of 0.982, suggesting that *ORC6* overexpression could be highly indicative or significant valuable for diagnosing LUAD (Fig. 1F). Next, clinical prediction model line chart was drawn from TCGA-LUAD *ORC6* expression data. Based on the staging of LUAD patients, their age, smoking status, and the level of ORC6 expression, a total score was calculated by adding the scores of each individual. This total score establishes the probability of predicting the occurrence of 1-year, 3-year, and 5-year survival rates. The results indicate that higher ORC6 expression suggests a lower probability of survival (Fig. 1G). Kaplan–Meier survival analysis revealed a correlation between high *ORC6* expression and poor prognosis among LUAD patients (Fig. 1H). Moreover, high ORC6 expression correlates with poor survival specifically in LUAD patients aged over 65 (Fig. 1I), in female (but not male) LUAD patients (Fig. 1J, K), and in patients with both M1 and M0 LUAD (Fig. 1L). The lack of correlation between high ORC6 expression and poor survival in male LUAD patients (Fig. 1K) may stem from various factors such as biological differences, tumor microenvironment variations, treatment responses, or genetic and hormonal influences specific to males. Further investigation is certainly needed to understand the precise mechanisms driving these disparities.

Interestingly, *ORC6* expression is also significantly elevated in LUSC tissues (“Tumor”), when compared to that in normal lung tissues (“Normal”) (Fig. 1M). Moreover, *ORC6* expression in LUSC tissues is significantly higher than that in matched adjacent normal lung tissues (Fig. 1N). These bioinformatics studies reveal that ORC6 expression is elevated in NSCLC.

### ORC6 expression is elevated in NSCLC tissues of locally-treated patients

**Fig. 2:**
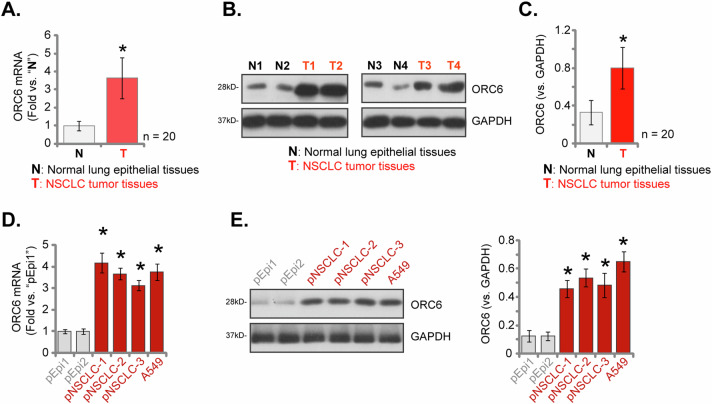
ORC6 expression is elevated in NSCLC tissues of locally treated patients. *ORC6* mRNA and protein expression profiles were shown from the NSCLC tumor tissues (“T”) and corresponding adjacent normal lung epithelial tissues (“N”) obtained from twenty primary NSCLC patients (**A**–**C**). The expression of *ORC6* mRNA and protein in the described NSCLC cells and lung epithelial cells was shown (**D**, **E**). The numerical values were the mean ± standard deviation (SD). Statistical significance was indicated by *P < 0.05 compared to “N” tissues or “pEpi1” cells.

Subsequently, we conducted an assessment of *ORC6* expression within localized NSCLC tissues. We obtained NSCLC tumor tissues (“T”) and corresponding adjacent normal lung epithelial tissues (“N”) from a cohort of twenty (*n* = 20) primary NSCLC patients (LUAD, stage III-IV). Examination of tissue lysates verified a substantial increase in *ORC6* mRNA expression within NSCLC tumor tissues compared to normal lung epithelial tissues (Fig. 2A). Additionally, the protein expression of ORC6 exhibited an upregulation in NSCLC tumor tissues from four selected patients (“T1” to “T4”) (Fig. 2B). Combining all 20 sets of tissue data, the collective analysis demonstrated a significant upregulation in ORC6 protein expression in NSCLC tumor tissues (Fig. 2C). Subsequent experiments were conducted to investigate the expression of ORC6 across different NSCLC cells, encompassing primary human NSCLC cells (“pNSCLC-1/2/3” derived from three distinct patients, as reported previously [34, 35]) and the immortalized A549 cells. The findings revealed a substantial elevation in *ORC6* mRNA expression within both primary and immortalized NSCLC cells, as compared to primary human lung epithelial cells (“pEpi1” and “pEpi2”, from two donors [37]) (Fig. 2D). Furthermore, an upregulation of ORC6 protein was observed in these different NSCLC cell lines (Fig. 2E), contrasting with the lower expression detected in the lung epithelial cells (Fig. 2E). These results provide further support for the increased expression of ORC6 in NSCLC tissues and cells.

### Bioinformatics analysis predicts the functional roles of ORC6 in cancer

**Fig. 3:**
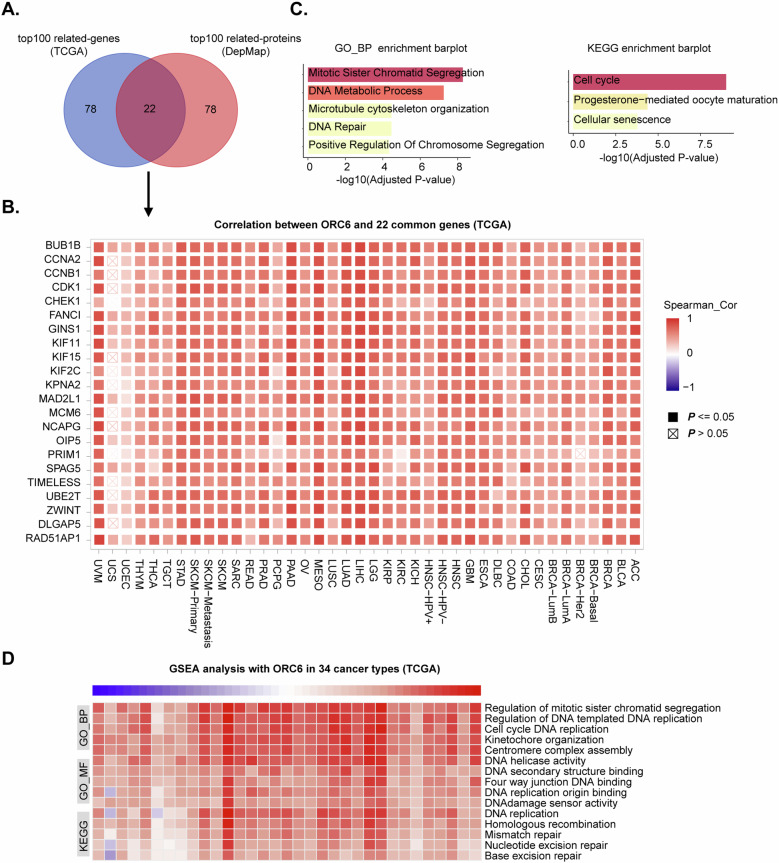
Bioinformatics analysis predicts the functional roles of ORC6 in cancer. The top 100 ORC6-related genes (from TCGA) and proteins (from DepMap) were obtained from pan-cancer databases, and the 22 shared parts were displayed in Venn diagram (**A**) and heat map (**B**). GO and KEGG analysis results of the shared parts were shown (**C**). Gene set enrichment analysis (GSEA) of biological processes (GO-BP), molecular functions (GO-MF), and KEGG pathway (KEGG) closely associated with ORC6 in 34 cancer types from TCGA database were shown (**D**).

Next, we conducted a comprehensive analysis to understand the possible functional roles of ORC6 in pan-cancer studies (34 cancer types). We utilized TCGA data to identify the top 100 ORC6-related genes and DepMap data to determine the top 100 ORC6-related proteins (Fig. 3A). A total of 22 common elements between the top 100 ORC6-related genes and proteins were identified (Fig. 3A, B). The Gene Ontology (GO) and Kyoto Encyclopedia of Genes and Genomes (KEGG) analyses on the 22 common parts revealed significant associations of ORC6 with mitosis, DNA synthesis, DNA repair, and cell cycle progression (Fig. 3C). Moreover, TCGA pan-cancer gene set enrichment analysis (GSEA) indicated a strong and positive correlation between ORC6 expression and mitosis, cell cycle progression, DNA repair and replication (Fig. 3D). The results collectively suggest that ORC6 is predicted to play a significant role in essential cellular processes such as mitosis, DNA synthesis, DNA repair, and cell cycle progression across various cancer types.

### ORC6 shRNA hinders viability, proliferation, cell cycle progression and migration in NSCLC cells

**Fig. 4:**
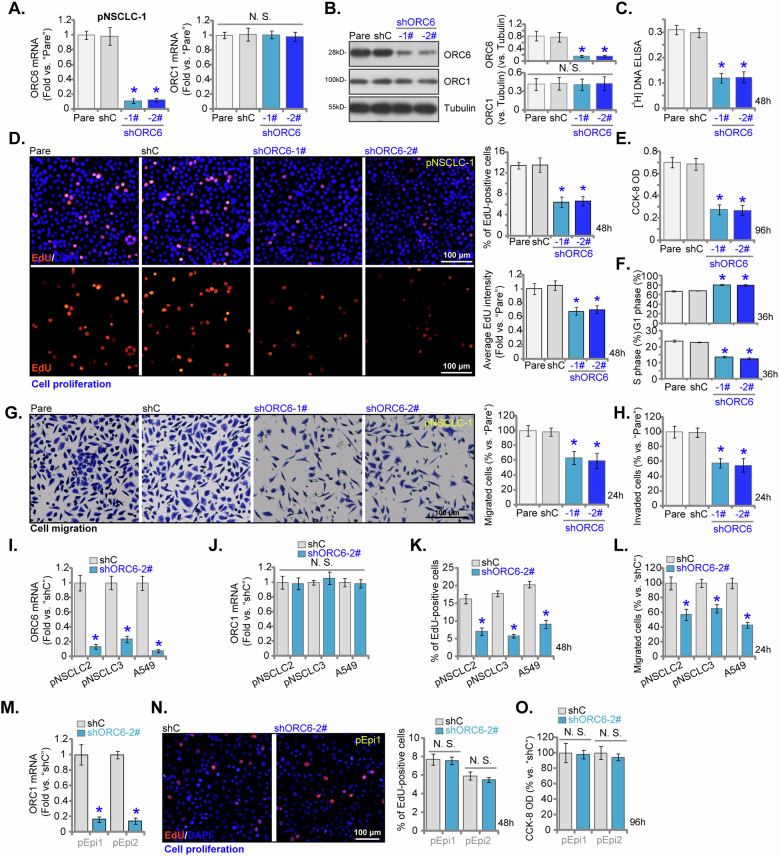
ORC6 shRNA hinders viability, proliferation, cell cycle progression and migration in NSCLC cells. The primary pNSCLC-1 cells were separately treated with specific ORC6 shRNAs (shORC6-1# and shORC6-2#, representing distinct sequences) or a control scramble non-sense shRNA (“shC”). The expression levels of ORC1/6 (both mRNA and protein) were assessed (**A**, **B**). Equal numbers of these cells were cultured for specific durations to evaluate various cellular functions, including [^3^H] DNA incorporation (**C**), cell proliferation (measuring the percentage of EdU-positive cells and the fluorescence intensity of EdU in each positive nucleus, **D**), viability (CCK-8 OD, **E**), cell cycle progression (**F**), and in vitro cell migration (“Transwell” assays, **G**) and invasion (“Matrigel Transwell”, **H**), with results quantifie. Furthermore, stable cells derived from other primary NSCLC cells (pNSCLC-2 and pNSCLC-3) or, the A549 immortalized cells, or the lung epithelial cells (“pEpi1” or “pEpi2”), expressing either shC or shORC6-2#, were established and examined for *ORC6* and *ORC1* mRNA expression (**I**, **J**, **M**). Equal numbers of these cells were cultured for specific durations to assess cell proliferation (**K**, **N**), migration (**L**) and viability (**O**) using the same methods. The numerical values were the mean ± standard deviation (SD, *n* = 5). “Pare” denotes parental control cells. Statistical significance was indicated by *P < 0.05 compared to “shC” cells, while “N. S.” signifies non-statistically significant differences (*P* > 0.05). The experiments depicted in this figure were repeated five times, yielding consistent results. Scale Bar = 100 μm.

To unveil the functional significance of ORC6 in NSCLC cells, a strategy employing shRNA was employed to knock down its expression. Specifically, lentivirus carrying ORC6 shRNA was introduced to the pNSCLC-1 primary NSCLC cells (as reported previously [34–36]), leading to the establishment of stable cells post-treatment with puromycin. Two distinct shRNAs targeting ORC6, shORC6-1# and shORC6-2# (with non-overlapping sequences), were utilized, both resulting in significant suppression of *ORC6* mRNA (Fig. 4A) and protein levels (Fig. 4B). The expression of ORC1 remained unaltered (Fig. 4A, B). The knockdown of ORC6 using these targeted shRNAs impeded the proliferation of pNSCLC-1 cells and hindered [^3^H] DNA incorporation (Fig. 4C). In addition, ORC6 shRNA not only reduced the percentage of EdU-positive pNSCLC-1 cells, but also significantly decreased the fluorescence intensity of EdU (“brightness”) in individual positive nuclei (Fig. 4D). Moreover, cell viability, assessed through CCK-8 optical density (OD), was significantly reduced in ORC6-silenced pNSCLC-1 primary cells (Fig. 4E). Additionally, silencing ORC6 in pNSCLC-1 cells disrupted the progression of the cell cycle (Fig. 4F), resulting in an increased percentage of cells in the G1 phase and a decreased percentage in the S phase (Fig. 4F). Furthermore, the employed ORC6 shRNAs demonstrated inhibitory effects on the mobility of pNSCLC-1 cells. The migration (Fig. 4G) and invasion (Fig. 4H) capabilities, evaluated through “Transwell” and “Matrigel Transwell” assays respectively, were significantly reduced following ORC6 shRNA treatment. Importantly, the scramble non-sense control shRNA (“shC”) did not induce significant alterations in ORC1/6 expression (Fig. 4A, B) or the functional consequence of pNSCLC-1 cells (Fig. 4C–H).

To investigate the consistency of ORC6 silencing across various primary NSCLC cells, the lentivirus expressing shORC6-2# was introduced into primary human NSCLC cells derived from different patients, namely, pNSCLC-2, pNSCLC-3, as well as into the A549 immortalized cells (see Fig. 2). The selection of stable cells was facilitated once again using puromycin. Results indicated a significant reduction in *ORC6* mRNA expression in these NSCLC cells with shORC6-2# (Fig. 4I), while *ORC1* mRNA expression remained unchanged (Fig. 4J). Consistent with the findings in pNSCLC-1 cells, the knockdown of ORC6 using shORC6-2# exhibited similar effects across these primary and immortalized NSCLC cells, suppressing cell proliferation (as evidenced by reduced EdU-positive cell percentage, Fig. 4K) and inhibiting cell migration (Fig. 4L).

In primary human lung epithelial cells, pEpi1 and pEpi2, the shORC6-2# method was employed to suppress ORC6 expression (Fig. 4M). However, the results indicate that despite ORC6 knockdown, there was no observable impact on cell proliferation (by measuring EdU-positive cell percentage) within the lung epithelial cells (Fig. 4N). Additionally, the assessment of cell viability using the CCK-8 method showed no significant inhibition caused by shORC6-2# (Fig. 4O).

### ORC6 shRNA causes apoptosis activation in NSCLC cells

**Fig. 5:**
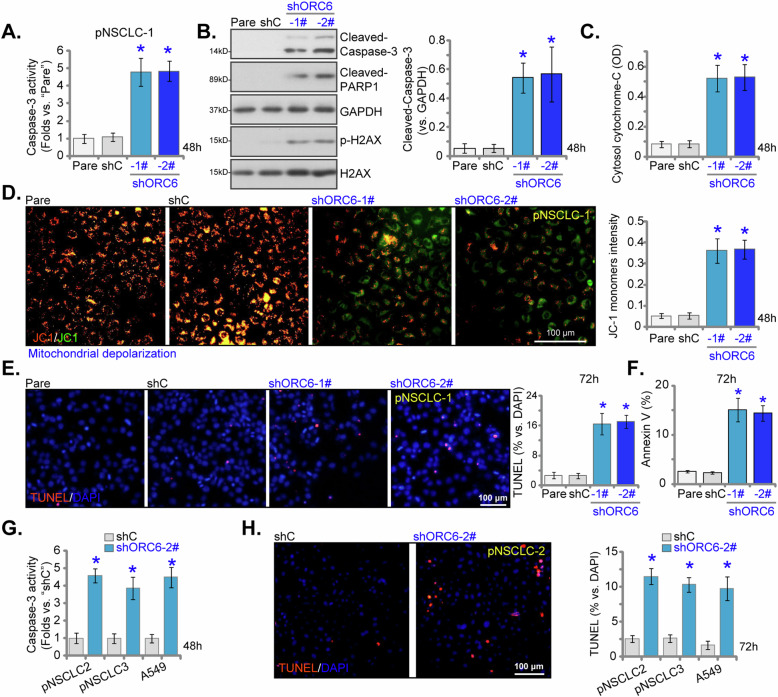
ORC6 shRNA causes apoptosis activation in NSCLC cells. The primary pNSCLC-1 cells were separately treated with specific ORC6 shRNAs (shORC6-1# and shORC6-2#, representing distinct sequences) or a control scramble non-sense shRNA (“shC”). Equal numbers of these cells were cultured for specific durations. The assessment included measuring Caspase-3 activity (**A**), cleavage of apoptosis-related proteins (**B**), cytosolic cytochrome C contents (ELISA, **C**), and mitochondrial depolarization indicated by the accumulation of JC-1 green fluorescence monomers (**D**). Cell apoptosis was evaluated by determining the percentage of nuclei incorporating TUNEL staining (**E**) or through recoding the percentage of cells with positive Annexin V staining in FACS assays (**F**). Stable cells derived from other primary NSCLC cells (pNSCLC-2 and pNSCLC-3) or the A549 immortalized cells, expressing either shC or shORC6-2#, were established and cultured for indicated time periods, Caspase-3 activity (**G**) and cell apoptosis (determining the percentage of nuclei incorporating TUNEL staining, **H**) were examined. The numerical values were the mean ± standard deviation (SD, *n* = 5). “Pare” denotes parental control cells. Statistical significance was indicated by *P < 0.05 compared to “shC” cells. The experiments depicted in this figure were repeated five times, yielding consistent results. Scale Bar = 100 μm.

shRNA-mediated knockdown of ORC6 exerted potent inhibitory effects on cell viability, proliferation, cell cycle progression, and migration in primary and immortalized NSCLC cells. To further investigate the functional role, we evaluated its influence on cell apoptosis. pNSCLC-1 cells treated with shORC6-1# or shORC6-2# exhibited a significant increase in Caspase-3 activity (Fig. 5A). The induced knockdown of ORC6 by shRNA also led to cleavages of Caspase-3 and PARP (−1) in these cells (Fig. 5B). Levels of phosphorylated H2AX (p-H2AX) were also elevated in ORC6-silenced pNSCLC-1 cells, providing evidence of DNA damage (Fig. 5B). Additionally, the cytosolic cytochrome-C levels, a key indicator of apoptosis activation, were elevated in ORC6-shRNA-expressing pNSCLC-1 cells (Fig. 5C). Furthermore, ORC6 silencing resulted in the accumulation of JC-1 green fluorescence monomers in pNSCLC-1 cells, indicative of mitochondrial depolarization (Fig. 5D). This ORC6 silencing-induced cascade ultimately led to increased apoptosis in pNSCLC-1 cells, evident from the significant increase in nuclei with positive TUNEL staining (Fig. 5E) and the increased percentage of Annexin V-stained pNSCLC-1 cells (Fig. 5F). As anticipated, the shC control treatment failed to induce Caspase-apoptosis activation in pNSCLC-1 cells (Fig. 5A–F). In other primary human NSCLC cells (pNSCLC-2 and pNSCLC-3), as well as in immortalized A549 cells, the establishment of stable ORC6 knockdown using the shORC6-2#-expressing lentivirus (see Fig. 4) similarly triggered Caspase-3 activation (Fig. 5G) and increased the count of nuclei displaying positive TUNEL staining (Fig. 5H), supporting apoptosis activation.

### ORC6 KO exerts profound anti-NSCLC cell activity

**Fig. 6:**
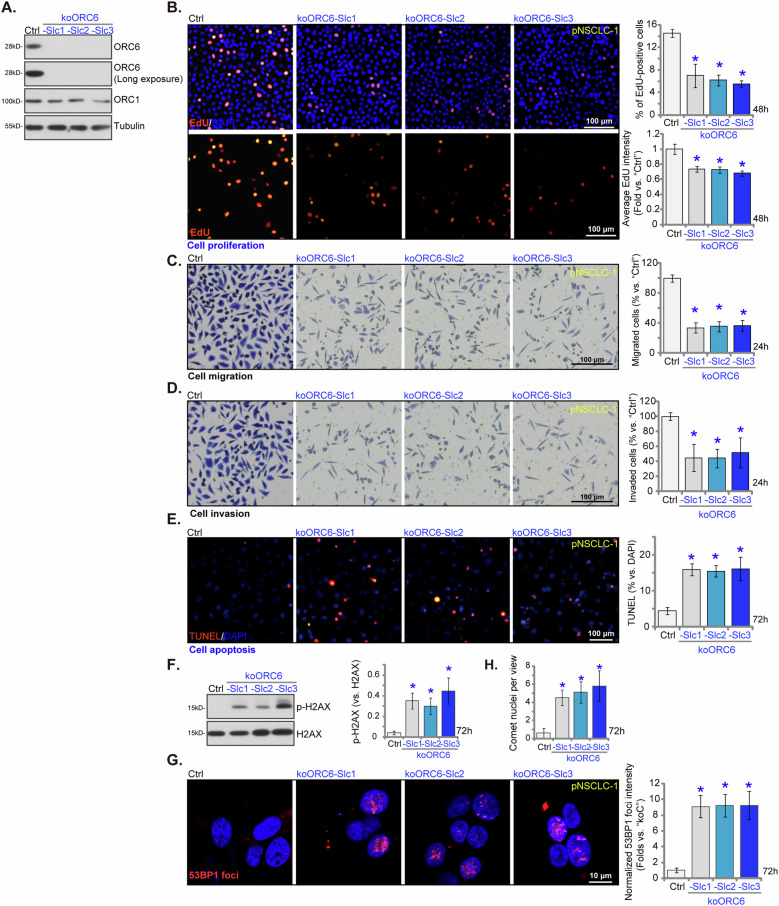
ORC6 KO exerts profound anti-NSCLC cell activity. The “koORC6” pNSCLC-1 cells, koORC6-Slc1, koORC6-Slc2, and koORC6-Slc3 (three sub-clones), were generated by introducing the CRISPR-ORC6-KO construct into Cas9-expressing pNSCLC-1 cells. Control cells, labeled as “Ctrl”, were established by combining the Cas9-expressing construct with the CRISPR-KO control construct. The protein expression of ORC6 and ORC1 was shown (**A**). Equal numbers of these cells were cultured for specific durations to evaluate various cellular functions, including cell proliferation (measuring the percentage of EdU-positive cells and the fluorescence intensity of EdU in each positive nucleus, **B**), in vitro cell migration (“Transwell” assays, **C**) and invasion (“Matrigel Transwell”, **D**), as well as cell apoptosis (by measuring nuclear TUNEL staining, **E**); DNA damage was tested via measuring phosphorylated H2AX (**F**), nuclear 53BP1 foci (**G**) and average Comet nuclei per microscope view (**H**). The numerical values were the mean ± standard deviation (SD, *n* = 5). Statistical significance was indicated by *P < 0.05 compared to “Ctrl” cells. The experiments depicted in this figure were repeated five times, yielding consistent results. Scale Bar = 100/10 μm.

To eliminate potential off-target effects caused by applied shRNAs and achieve a complete KO of ORC6, the CRISPR/Cas9 strategy was utilized. Specifically, the CRISPR/Cas9-ORC6-KO puro-construct-expressing lentivirus was introduced to Cas9-expressing pNSCLC-1 cells (reported previously [34]). Puromycin selection yielded single stable cells, subjected to *ORC6* KO screening, resulting in the establishment of single-cell-derived NSCLC-1 ORC6 KO colonies: koORC6-Slc1, koORC6-Slc2, and koORC6-Slc3. Immunoblotting analysis demonstrated a depletion of ORC6 protein expression in koORC6 pNSCLC-1 cells (Fig. 6A). ORC6 expression was however intact in pNSCLC-1 cells harboring the CRISPR/Cas9 control construct (“Ctrl”) (Fig. 6A). The expression of ORC1 remained unaltered in koORC6 pNSCLC-1 cells (Fig. 6A). Functional assays revealed substantial inhibitory effects of ORC6 KO on pNSCLC-1 cell proliferation. ORC6 KO decreased the percentage of EdU-positive pNSCLC-1 cells and inhibited the fluorescence intensity of EdU in individual positive nuclei (Fig. 6B). Additionally, in vitro cell migration (Fig. 6C) and invasion (Fig. 6D) of the koORC6-Slc1, koORC6-Slc2, and koORC6-Slc3 pNSCLC-1 cells were inhibited. ORC6 KO also induced apoptosis activation in the pNSCLC-1 cells, evidenced by the increased nuclear TUNEL ratio (Fig. 6E). Furthermore, ORC6 KO induced DNA damage in NSCLC cells. This is evidenced by the significant increase in the levels of phosphorylated H2AX (p-H2AX, Fig. 6F) and 53BP1 foci (Fig. 6G) in koORC6-Slc1, koORC6-Slc2, and koORC6-Slc3 pNSCLC-1 cells. Additionally, the quantified Comet assay results further confirmed substantial DNA damage in the ORC6 KO pNSCLC-1 cells, where Comet nuclei (per view) were increased (Fig. 6H).

### ORC6 overexpression augments proliferation and migration of NSCLC cells

**Fig. 7:**
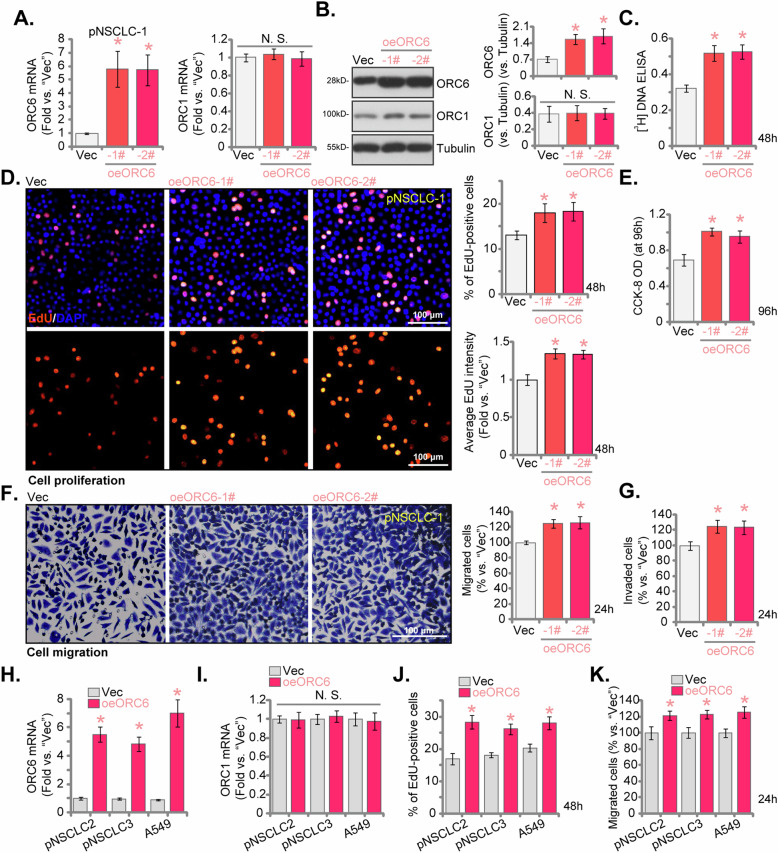
ORC6 overexpression augments proliferation and migration of NSCLC cells. pNSCLC-1 cells expressing the ORC6 construct, specifically “oeORC6-1#” and “oeORC6-2#” (representing two stable cell selections), along with those containing the empty vector (“Vec”), were generated for analysis of ORC6 and ORC1 expression at both mRNA and protein levels (**A**, **B**). Equal numbers of these cells were cultured for specific durations to evaluate various cellular functions, including [^3^H] DNA incorporation (**C**), cell proliferation (measuring the percentage of EdU-positive cells and the fluorescence intensity of EdU in each positive nucleus, **D**) and viability (CCK-8 OD, **E**) as well as in vitro cell migration (“Transwell” assays, **F**) and invasion (“Matrigel Transwell”, **G**). Stable cells derived from other primary NSCLC cells (pNSCLC-2 and pNSCLC-3) or the A549 immortalized cells, expressing either the ORC6 construct (“oeORC6”) or the empty vector (“Vec”), were established and were examined for *ORC6* and *ORC1* mRNA expression (**H**, **I**). Equal numbers of these cells were cultured for specific durations to assess cell proliferation (**J**) and migration (**K**) using the same methods. The numerical values were the mean ± standard deviation (SD, *n* = 5). Statistical significance was indicated by *P < 0.05 compared to “Vec” cells, while “N. S.” signifies non-statistically significant differences (*P* > 0.05). The experiments depicted in this figure were repeated five times, yielding consistent results. Scale Bar = 100 μm.

The outcomes presented above clearly demonstrate the substantial anti-cancer effect following the silencing or KO of ORC6 in both primary and immortalized NSCLC cells. This led us to speculate that ectopic ORC6 overexpression might yield contrasting effects, potentially fostering the growth of NSCLC cells. Consequently, the lentivirus containing the ORC6-expressing construct (“oeORC6”) were introduced to pNSCLC-1 cells. After subjecting the cells to puromycin treatment, we obtained two stable cell lines, namely “oeORC6-1#” and “oeORC6-2#”. Comparing with the control pNSCLC-1 cells carrying an empty vector (“Vec”), a remarkable upregulation in both *ORC6* mRNA (Fig. 7A) and protein expression (Fig. 7B) was detected in oeORC6 cells. The expression of ORC1, however, remained unaltered (Fig. 7A, B). The proliferation of pNSCLC-1 cells was significantly augmented upon ORC6 overexpression. This was evidenced by an increase in [^3^H] DNA incorporation (Fig. 7C). Moreover, ectopic overexpression of ORC6 increased the percentage of EdU-positive pNSCLC-1 cells and enhanced the fluorescence intensity of EdU in each positive nucleus (Fig. 7D). Furthermore, oeORC6 substantially enhanced CCK-8 viability in pNSCLC-1 cells (Fig. 7E). The ectopic overexpression of ORC6 also expedited in vitro cell migration (Fig. 7F) and invasion (Fig. 7G) of pNSCLC-1 cells.

Subsequently, the lentivirus carrying the ORC6-expressing construct (“oeORC6”) was introduced to additional primary NSCLC cells (pNSCLC-2/pNSCLC-3) as well as the immortalized A549 cells, resulting in the formation of stable cells post-puromycin selection. In these NSCLC cells, there was a notable increase in *ORC6* mRNA levels (Fig. 7H), while *ORC1* mRNA remained unchanged (Fig. 7I). The overexpression of ORC6 amplified proliferation by enhancing the percentage of EdU-positive NSCLC cells (Fig. 7J). Furthermore, ORC6 overexpression led to an accelerated in vitro cell migration in the primary and immortalized NSCLC cells (Fig. 7K). These results further supported a pivotal role of ORC6 in the progression of NSCLC.

### ORC6 is important for expression of multiple cyclins in NSCLC cells

**Fig. 8:**
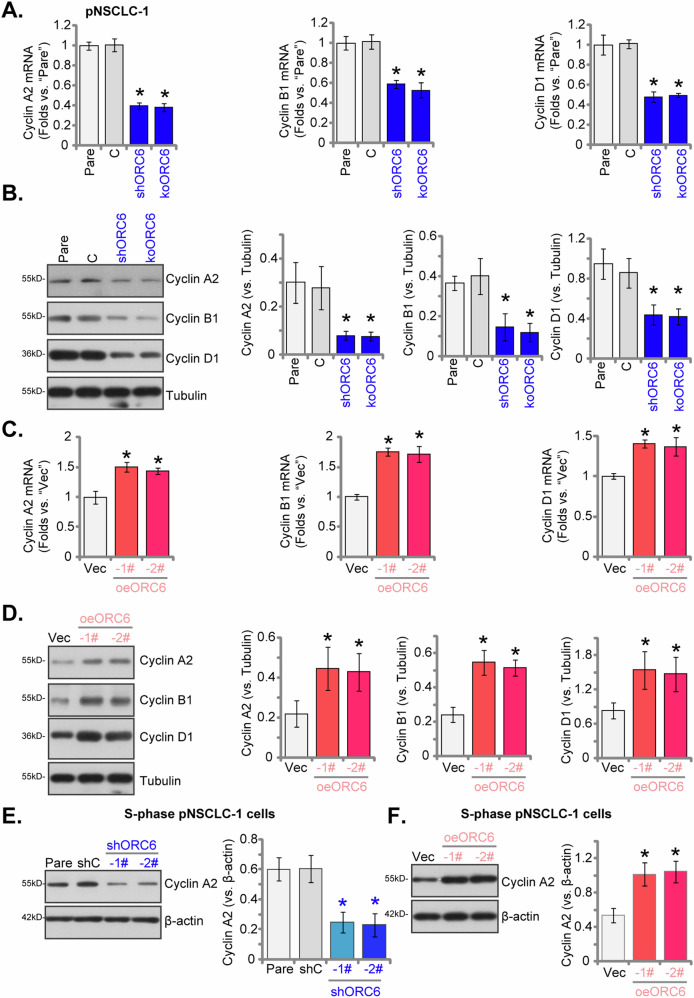
ORC6 is important for expression of multiple cyclins in NSCLC cells. The mRNA and protein expression of listed cyclins in the pNSCLC-1 cells with shORC6-2# (“shORC6”), the CRISPR-ORC6-KO construct plus the Cas9-expressing construct (“koORC6”), or the scramble non-sense control shRNA combined with the CRISPR/Cas9 control construct (“C”) was shown (**A**, **B**). The mRNA and protein expression of listed cyclins in pNSCLC-1 cells expressing the ORC6 construct (“oeORC6-1#” and “oeORC6-2#”, representing two stable cell selections) or the empty vector (“Vec”) was shown (**C**, **D**). The primary pNSCLC-1 cells were separately treated with specific ORC6 shRNAs (shORC6-1# and shORC6-2#, representing distinct sequences) or a control scramble non-sense shRNA (“shC”). Equal numbers of these cells were stained with PI and EdU, with S-phase cells sorted via FACS. The expression of cyclin A2 was tested via Western blotting assays (**E**, **F**). The numerical values were the mean ± standard deviation (SD, *n* = 5). “Pare” denotes parental control cells. Statistical significance was indicated by *P < 0.05 compared to “C”/“Vec” cells. The experiments depicted in this figure were repeated five times, yielding consistent results.

The bioinformatics studies reveals that ORC6 could play a significant role in essential cellular process including mitosis, DNA synthesis and cell cycle progression in cancer. Among the 22 common genes/proteins closely associated with ORC6 are multiple cyclins, including Cyclin A2 and Cyclin B1. We therefore tested whether ORC6 is important for the expression of cyclins in NSCLC cells. In pNSCLC-1 cells, both silencing of ORC6 using shORC6-2# (“shORC6,” see Figs. 4 and 5) and ORC6 KO via the CRISPR/Cas9 strategy (“koORC6,” as shown in Fig. 6) resulted in a significant decrease in the mRNA expression of several key cyclins, including *Cyclin A2, Cyclin B1*, and *Cyclin D1* (Fig. 8A). Moreover, protein expression of Cyclin A2, Cyclin B1 and Cyclin D1 was downregulated as well in shORC6 and koORC6 pNSCLC-1 cells (Fig. 8B). In contrast, the control treatment (“C”), which involved the use of the scramble non-sense control shRNA combined with the CRISPR/Cas9 control construct, did not induce any changes in the expression of these cyclins in pNSCLC-1 cells (Fig. 8A, B). Interestingly, in pNSCLC-1 cells overexpressing ORC6 (“oeORC6-1#” and “oeORC6-2#”, detailed in Fig. 7), both mRNA and protein levels of the aforementioned cyclins were significantly increased (Fig. 8C, D). Changes in the cell cycle profiles, such as a decrease in S-phase cells, may potentially impact the expression levels of cyclins. Our study revealed a decrease in cyclin A2 levels in ORC6-silenced S-phase pNSCLC-1 cells (Fig. 8E), whereas an increase was observed in ORC6-overexpressed S-phase pNSCLC-1 cells (Fig. 8F). These findings provide support for the significance of ORC6 in regulating the expression of multiple cyclins in NSCLC cells.

### ORC6 silencing hinders primary NSCLC cell xenograft growth in nude mice

Aiming to investigate the potential role of ORC6 on NSCLC cell growth in vivo, the primary pNSCLC-1 cells, at a count of six million cells per mouse, were subcutaneously injected into the flanks of nude mice. Subsequently, pNSCLC-1 xenografts were established within 3 weeks, reaching a volume close to 100 mm^3^ (“day-0”). Following this, nude mice bearing the pNSCLC-1 xenografts were randomly divided into two groups. The treatment group received intratumoral injections of adeno-associated virus (aav)-packed shORC6-2# (“aav-shORC6-2#”), while the control group received intratumoral injections of aav-packed scramble control shRNA (“aav-shC”). Virus injections were repeated after 72 h (“day-3”), and tumor volumes were measured every five days.

**Fig. 9:**
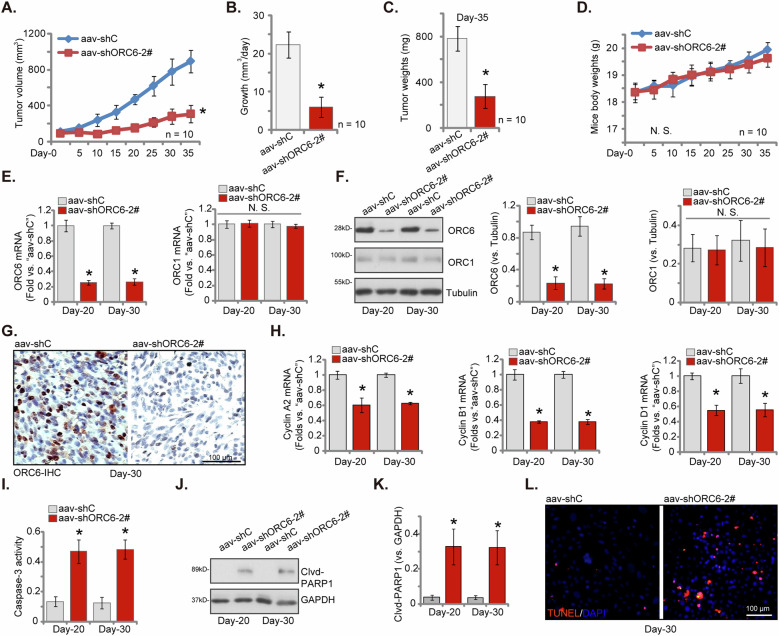
ORC6 silencing hinders primary NSCLC cell xenograft growth in nude mice. Nude mice carrying pNSCLC-1 xenografts were treated by intratumoral injection with adeno-associated virus expressing shORC6-2# (“aav-shORC6-2#”) or a control shRNA adeno-associated virus (“aav-shC”). The volumes of the pNSCLC-1 xenografts (**A**) and the body weights of the animals (**D**) were monitored at five-day intervals. The daily growth rate of the pNSCLC-1 xenografts was calculated (**B**). At day-35, all pNSCLC-1 xenografts were excised and weighed (**C**). Tissue lysates from these xenografts were analyzed for the expression of specific mRNAs and proteins as listed (**E**, **F**, **H**, **J**, **K**), and the Caspase-3 activity was also assessed (**I**). Alternatively, sections of the pNSCLC-1 xenografts were subjected to IHC staining for ORC6 (**G**), or they underwent immuno-fluorescence staining for TUNEL/DAPI (**L**). The numerical values were the mean ± standard deviation (SD). Statistical significance was indicated by *P < 0.05 compared to “aav-shC” treatment, while “N. S.” signifies non-statistically significant differences (***P*** > 0.05). For **A**–**D**, each group consisted of ten mice (*n* = 10). In **E**–**K**, five random tissue samples from each xenograft were analyzed (*n* = 5). Scale Bar = 100 μm.

The injection of aav-shORC6-2# significantly hindered the growth of pNSCLC-1 xenografts in nude mice, leading to substantially reduced xenograft volumes (Fig. 9A). Employing the described approach [34, 35], the daily estimated growth of pNSCLC-1 xenografts, measured in mm^3^ per day, was computed (Fig. 9B). The results clearly indicated a significant suppression of pNSCLC-1 xenograft growth following aav-shORC6-2# injection (Fig. 9B). The animal experiments concluded at day-35, and all pNSCLC-1 xenografts were isolated and weighed. The pNSCLC-1 xenografts from the aav-shORC6-2# group were substantially lighter and smaller than those from the aav-shC group (as depicted in Fig. 9C). Conversely, the body weights of the animals showed no significant differences between the groups (Fig. 9D). These findings provide strong evidence supporting the inhibitory effect of aav-shORC6-2# injection on NSCLC xenograft growth in nude mice.

On the 20th day (day-20) and the 30th day (day-30) subsequent to the first virus injection, a single pNSCLC-1 xenograft was extracted from each group, resulting in a total of four xenografts. Part of these xenografts were dissected and homogenized. The findings revealed a significant decrease in both mRNA (Fig. 9E) and protein (Fig. 9F) expression levels of ORC6 within the tissues of pNSCLC-1 xenografts treated with aav-shORC6-2#. In contrast, the expression of ORC1 remained unchanged (Fig. 9E, F). The results of the immunohistochemistry (IHC) assay further corroborated the downregulation of ORC6 (a nuclear protein) in the pNSCLC-1 xenograft tissues treated with aav-shORC6-2# (see Fig. 9G).

Importantly, the mRNA expression of several key cyclins, including *Cyclin A2*, *Cyclin B1* and *Cyclin D1*, was significantly downregulated in aav-shORC6-2#-treated pNSCLC-1 xenograft tissues (Fig. 9H). Further analysis of the xenograft tissues revealed an increase in Caspase-3 activity in pNSCLC-1 xenograft tissues with aav-shORC6-2# injection (Fig. 9I), which was accompanied by elevated levels of cleaved-PARP1 (Fig. 9J, K). Furthermore, findings from immunofluorescence analysis of xenograft sections corroborated the induction of apoptosis in ORC6-silenced pNSCLC-1 xenografts. This was evidenced by a heightened percentage of TUNEL-positive apoptotic nuclei (Fig. 9L). Collectively, these findings indicated that injection of aav-shORC6-2# led to silencing of ORC6, downregulation of cyclins, and activation of apoptosis in pNSCLC-1 xenografts.

## Discussion

Targeted therapies in NSCLC, including inhibitors of EGFR, ALK, ROS1 and MET [9, 10, 14], exhibit remarkable precision but face limitations such as the emergence of drug resistance over time, reliance on specific biomarkers for efficacy, potential side effects, limited long-term effectiveness, and challenges related to cost and accessibility [38, 39]. Ongoing research endeavors aim to overcome these hurdles by exploring alternative targets, pivotal in advancing the efficacy and accessibility of targeted therapies for NSCLC.

ORC6 is a protein involved in DNA replication and cell cycle progression. Its primary function involves aiding the formation of pre-replication complexes at DNA replication origins, ensuring precise and efficient replication of DNA during cell cycle progression and cell division [25, 26, 28]. Its dysregulation correlates with aggressive tumor behavior and poorer patient outcomes in several cancers, making it a potential biomarker and therapeutic target [29, 31–33]. Reducing ORC6 levels heightened the sensitivity of colon cancer cells to 5-FU and cisplatin, causing cell cycle arrest, multi-nucleation, and activating p53-associated pathways, suggesting ORC6 as a promising target for colon cancer [33]. Pan et al. have also shown that ORC6 overexpression significantly correlated with shorter overall survival in clear cell renal cell carcinoma, serving as an independent prognostic factor [30]. It differentiated between tumor and normal patients, emphasizing its diagnostic potential [30].

Here, bioinformatics studies indicate a significant increase in *ORC6* expression in LUAD tissues, demonstrating a strong correlation with diminished overall survival. Additionally, the upregulation of ORC6 in LUAD exhibits a higher significance in patients with advanced M-stages/pathological stages, and among those deceased with the disease. *ORC6* mRNA and protein expression is also elevated in localized human NSCLC tissues and primary/immortalized NSCLC cells. Silencing or KO of ORC6 in these cells inhibited cell viability, proliferation, and migration, disrupted cell cycle progression and activated apoptosis. Conversely, ectopic ORC6 overexpression enhanced NSCLC cell proliferation and migration. Remarkably, intratumoral injection of ORC6 shRNA aav significantly impeded the growth of subcutaneous NSCLC xenografts in nude mice. Thus, ORC6 silencing/KO exhibited potent inhibition of NSCLC cell growth.

Cyclins, the key regulators of the cell cycle, play a crucial role in the pathogenesis and progression of NSCLC [40–42]. Dysregulation of cyclins and cyclin-dependent kinases (CDKs) disrupts the normal cell cycle progression, leading to uncontrolled cell proliferation and NSCLC growth [43, 44]. Various cyclins, such as cyclin D1 and cyclin B1, have been implicated in NSCLC progression. Elevated expression of cyclin D1, for example, has been associated with more aggressive behavior and poor prognosis in NSCLC patients [45]. These cyclins drive the cell cycle progression, promoting unchecked cell division and tumor growth [40–42]. Additionally, the abnormal expression of cyclins often correlates with genetic alterations or mutations in signaling pathways that control their expression, contributing to the development and progression of NSCLC [43, 44]. Understanding the mechanisms of cyclins dysregulation in NSCLC progression is crucial.

ORC6’s involvement in DNA replication, DNA damage response pathways, transcriptional regulation, and cell cycle progression suggests potential indirect effects on cyclins expression [25, 26, 28]. Changes in replication timing, DNA damage response, and transcriptional regulation due to ORC6 dysfunction could alter cyclin expression levels and cell cycle progression [25, 27, 31, 46]. We presented evidence supporting ORC6’s significance in regulating several cyclins within NSCLC. Bioinformatics studies highlighted ORC6’s potential pivotal role in cancer by influencing DNA synthesis and cell cycle progression. Among 22 closely linked genes of ORC6, multiple cyclins were identified. In primary NSCLC cells, silencing or KO of ORC6 led to reduced expression of Cyclin A2, Cyclin B1, and Cyclin D1, whereas their levels increased in ORC6-overexpressing NSCLC cells. Similarly, the expression of the above cyclins was also decreased in ORC6-silenced NSCLC xenograft tissues. Considering that depletion of ORC6 resulted in proliferation inhibition, cell cycle arrest, and apoptosis induction in cultured NSCLC cells and NSCLC xenografts, we conclude that modulating cyclin expression could be a fundamental mechanism for ORC6-driven NSCLC progression.

It’s important to acknowledge that changes in cell cycle distribution, as observed in the ORC6-depleted NSCLC cells, could influence the expression of cyclins (including cyclin A). Here we show that cyclin A2 levels were decreased in ORC6-silenced S-phase pNSCLC-1 cells, but increased in ORC6-overexpressed S-phase pNSCLC-1 cells. In our upcoming project, we’ll undertake a comprehensive investigation to understand how ORC6 modulation influences cyclins expression across various cell cycle profiles, and its consequent effects on cell proliferation and DNA replication in NSCLC cells.

In line with our findings, Chen et al. recently reported that elevated ORC6 expression is strongly correlated with poor clinical outcomes in NSCLC patients, independently contributing to overall survival risk [46]. Kaplan-Meier analysis also supported ORC6 as a valuable prognostic indicator [46].

In summary, ORC6, a crucial component of the origin recognition complex, is essential for DNA replication [26, 28, 47, 48]. Its depletion in NSCLC cells, achieved through shRNA or CRISPR/Cas9 techniques, leading to reduced DNA synthesis. It also resulted in DNA damage, evidenced by increased H2AX phosphorylation, formation of 53BP1 foci, and accumulation of Comet nuclei in ORC6 KO NSCLC cells. Such DNA damage shall subsequently trigger cell apoptosis. Moreover, the depletion of ORC6 also seems to reduce the expression of cyclins, further inhibiting NSCLC cell proliferation by disrupting cell cycle progression. A deeper understanding of these mechanisms could unveil significant therapeutic opportunities, suggesting that targeting ORC6 or its interactions could represent a promising strategy for NSCLC treatment.

## Supplementary information

SUPPLEMENTAL Figure 1

## Data Availability

All data are available upon reasonable request.
