# Supplementary material for: Elevated origin recognition complex subunit 6 expression promotes non-small cell lung cancer cell growth
Source: Cell Death Dis. 2024 Sep 30;15(9):700. doi: 10.1038/s41419-024-07081-y (PMC11442828; doi:10.1038/s41419-024-07081-y)
Supplement: Supplementary file 1 — SUPPLEMENTAL Figure 1 [file 41419_2024_7081_MOESM1_ESM.pdf]

Figure S1.

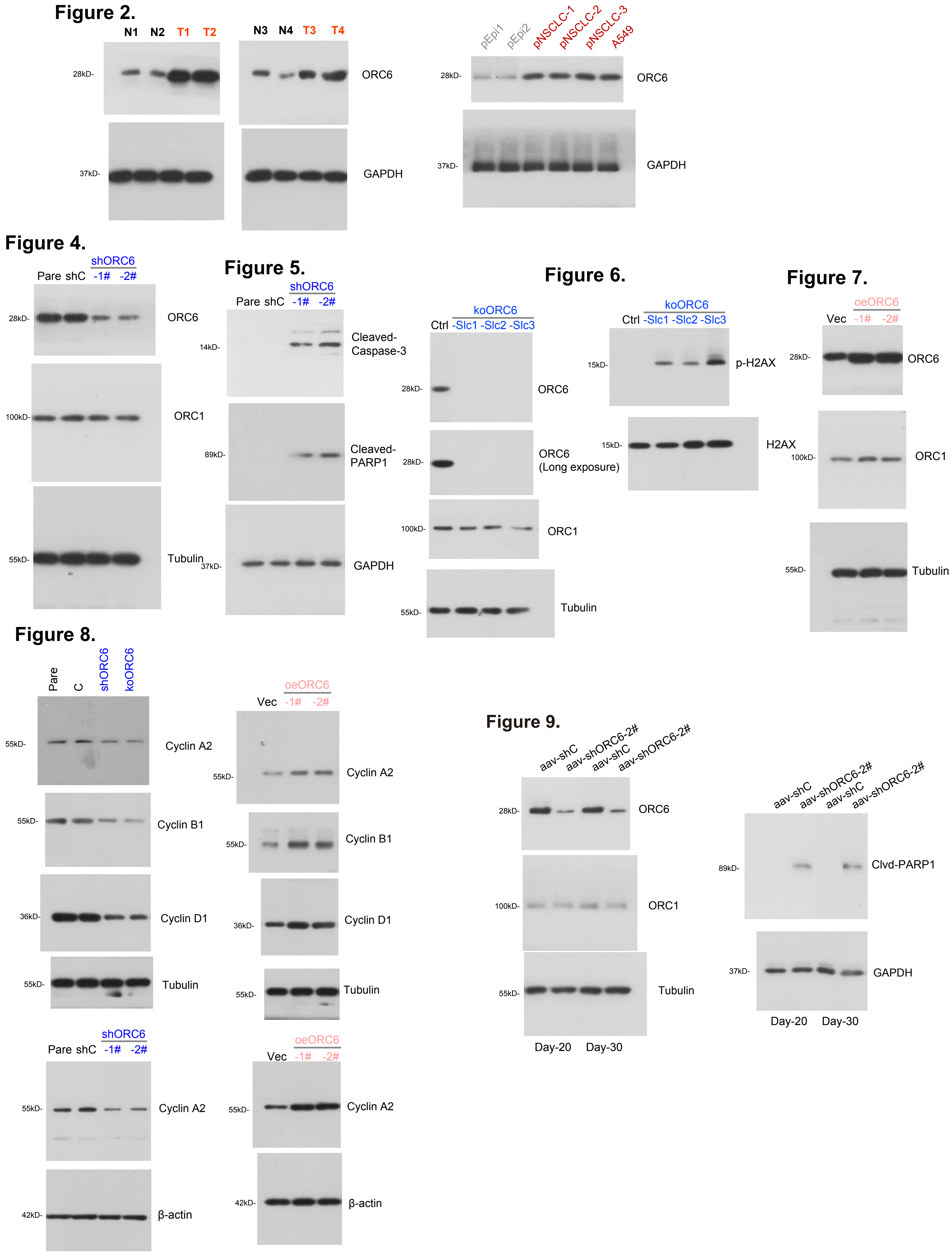


**Figure S1. The uncropped blotting images of the study.**
